# Improving the neural mechanisms of cognition through the pursuit of happiness

**DOI:** 10.3389/fnhum.2013.00452

**Published:** 2013-08-07

**Authors:** Karuna Subramaniam, Sophia Vinogradov

**Affiliations:** Department of Psychiatry, University of CaliforniaSan Francisco, San Francisco, CA, USA

**Keywords:** positive mood, creative cognition, medial prefrontal cortex, fMRI, real-time fMRI

## Abstract

This paper reviews evidence on the neural basis of how positive mood states can modulate cognition, particularly during creative problem-solving. Studies performed over the past few decades demonstrate that individuals in a positive mood engage in a broader scope of attention, enhancing their access to distant and unusual semantic associations, and increasing task-shifting and problem-solving capacities. In this review, we summarize these behavioral studies; we then present recent findings on the changes in brain activation patterns that are induced by a positive mood when participants engage in problem-solving tasks and show how these relate to task performance. Additionally, we integrate findings on the neuromodulatory influence of positive mood on cognition as mediated by dopaminergic signaling in the prefrontal cortex and we describe how this system can go awry during pathological states of elevated mood as in mania. Finally, we describe current and future research directions using psychotherapeutic and real-time fMRI neurofeedback approaches to up-regulate positive mood and facilitate optimal creative cognitive performance. We conclude with some speculations on the clinical implications of this emerging area of research.

## Introduction

Positive mood states—states of *happiness*—are the mental or emotional states of well-being characterized by positive emotions ranging from quiet contentment to intense joy (http://en.wikipedia.org/wiki/Happiness). When Abraham Lincoln said, “People are just as happy as they make up their minds to be,” he was suggesting that happiness is a state of mind which to a certain extent is under our control. This is an important point since happiness is arguably the single most important factor for enhancing life satisfaction and success (Fredrickson, 2000). Happiness has been shown to be both the cause and consequence of success across various life domains including marriage, friendship, work performance, and overall health (Lyubomirsky et al., 2005; Sin and Lyubomirsky, 2009). For example, happiness is associated with better social interactions and higher work incomes (Lyubomirsky et al., 2005); better coping abilities (Moskowitz, 2010); health-related benefits (Moskowitz et al., 2008); self-beliefs of enhanced health, intelligence and social interactions (Myers and Diener, 1995); and greater personal resilience and overall well-being (Fredrickson, 2000, 2004). People in happy, positive mood states are better able to modulate cognition by enhancing broader thought-action repertoires, and demonstrate broadened attention, greater cognitive flexibility, and heightened creativity (Isen et al., 1985, 1987, 1991; Estrada et al., 1994; Fredrickson, 2004; Isen, 2005). Taken together, within the past three decades, studies from the positive psychology literature have revealed a plethora of behavioral evidence demonstrating that positive mood states contribute to life satisfaction and enhance certain aspects of cognitive performance.

Although the behavioral evidence of the interactions between positive mood and cognition is well-documented, there are very few studies that demonstrate how a positive mood can influence cognition at the neural systems level (e.g., Subramaniam et al., 2009). A deeper understanding of these neurobiological processes is critical, not just for understanding these mechanisms in healthy individuals, but for broadening our approach to the development of treatments for individuals with psychiatric illnesses that are often characterized by deficits in positive mood and cognition, such as people suffering from depressive disorders and schizophrenia.

## Behavioral evidence demonstrates that positive mood modulates cognitive operations

Positive mood states facilitate a broad scope of attention (Gasper and Clore, 2002; Bolte et al., 2003) and enhance more integrative access to distant semantic associations (Isen et al., 1985; Federmeier et al., 2001; Friedman et al., 2003). This broader and more holistic mode of attention predicts and promotes cognitive control/flexibility, including flexible coping skills (Koestler, 1964; Aspinwall and Taylor, 1997) and creative solutions to problem-solving tasks (Isen et al., 1987; Rowe et al., 2007). Cognitive control is a multifaceted set of operations that includes the ability to inhibit dominant yet incorrect problem-solving paths and to switch between problem-solving strategies, and/or between broad and focused modes of attention. The result of increased cognitive control is that solvers are better able to select and act upon non-dominant yet correct solutions. A positive mood enhances these cognitive control processes by facilitating switching between broad and focused attentional modes (Baumann and Kuhl, 2005), and between different solving strategies (Dreisbach and Goschke, 2004), so that solvers are better able to reduce perseveration tendencies on errant problem-solving paths and increase selection of the correct solution (Ashby et al., 1999, 2002). Although the different studies described below induced positive mood in participants through various techniques, they all conducted objective mood manipulation measures to ensure that the target mood was induced and maintained. Together, the findings suggest that regardless of the mood induction method, a positive mood reliably enhances cognitive processes in distinct ways. We discuss each of these processes in more detail below.

### Positive mood facilitates a broadening scope of attention

**Figure 1:**
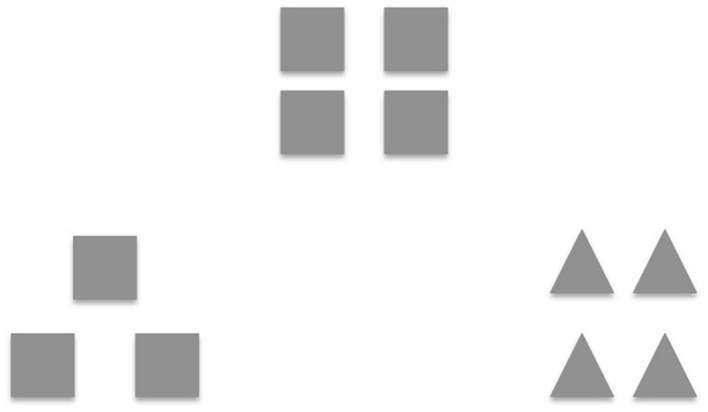
**Global-local task.** One item from the global-local shape task [adapted with permission from Kimchi and Palmer (1982)]. Participants had to indicate which one of two sample figures most resembled the target figure (i.e., square made of squares). Each figure was either a square or a triangle (global feature) made up of smaller squares or triangles (local feature).

Prior research suggests that a positive mood broadens the scope of attention, thoughts and actions (Fredrickson, 2001; Gasper and Clore, 2002; Bolte et al., 2003; Fredrickson and Branigan, 2005); this involves attending to more stimuli in both external visual space and internal semantic space, allowing access to more information to simultaneously influence solution efforts (Rowe et al., 2007). Furthermore, positive mood states are associated with greater global or holistic processing (i.e., seeing the forest before the trees) vs. local processing (i.e., seeing the trees before the forest). For example, Gasper and Clore (2002) induced positive and negative mood states by asking healthy participants to recall personal life events that made them feel either positive or negative. The mood manipulation check was effective; participants felt more positive and less negative after writing about a happy event. Subsequently, they measured mood-attention relationships with a visual matching test (see Figure 1, adapted from Kimchi and Palmer, 1982) in which participants were instructed to indicate which one of two sample figures looks most like a target figure. Each figure was either a square or a triangle (global feature) made up of smaller squares or triangles (local feature). Results revealed that positive mood participants were more likely to match the objects on the basis of global features than participants in a negative mood (Gasper and Clore, 2002). In a subsequent study, Fredrickson and Branigan (2005) replicated similar findings to the Gasper and Clore (2002), using an alternative mood induction technique of positive, neutral and negative film clips to induce the target mood state in healthy participants. When comparing participants' mood ratings to the film clips, Fredrickson and Branigan (2005) found that each film clip was effective at inducing the target mood state. They also found that after participants watched the positive films, they showed a significantly larger global bias on the global-local visual task when compared to the neutral mood participants, confirming their hypothesis that a positive mood broadens attention.

### Positive mood facilitates integration and access to distant semantic associations across a diverse range of tasks

Positive mood can also enhance integration processes, facilitating a broader access to remote semantic associations (Isen et al., 1987; Estrada et al., 1997; Federmeier et al., 2001; Bolte et al., 2003), which, in turn, facilitates creative problem solving. Some examples of how a positive mood enhances integration processes arise from word association tasks. Isen et al. (1985) used several different mood induction methods to induce the target mood state in participants: in one experiment, they used affectively-valenced (positive, neutral, and negative) words; in another experiment, they used positive and neutral film clips; and in a third experiment they gave participants candy vs. no manipulation. After each of these mood induction methods, participants performed word-association tasks in which they were presented with a neutral word and were then required to respond with word associations that came to mind (Isen et al., 1985; Ashby et al., 1999). Regardless of the mood induction method, Isen et al. (1985) found that participants in a positive mood were more likely to respond with remote word associations compared to participants in a neutral mood. For example, the dominant interpretation of the word *pen* is a writing tool; neutral-mood participants were likely to respond with a high frequency associate, such as *pencil* or *paper*. However, participants in a positive mood also included remote interpretations of *pen* as a fenced enclosure, and were thus more likely to respond with associations such as barn or pig. Thus, in Ashby et al.'s (1999) connectionist model of cognitive set selection, a positive mood led to the selection of a broader set of remote associations, which is known to promote creative solving ability.

These beneficial effects of a positive mood on enhancing integration processes across a broad set of semantic associations has been demonstrated many times, using a diverse range of tasks and populations—from college students doing word association and creative problem-solving tasks, to industrialists engaging in negotiations, to doctors integrating non-dominant yet relevant information earlier while solving medical cases (Carnevale and Isen, 1986; Isen et al., 1987; Estrada et al., 1994, 1997; Rowe et al., 2007). For example, when a positive mood was induced in doctors by giving them candy, they integrated non-dominant remote information earlier when solving a rare case of chronic hepatitis, as compared to controls in a neutral mood (Estrada et al., 1997). This positive mood-induced access to a broader range of associations and thoughts appears to facilitate cognitive control and creative problem-solving across a range of tasks (Isen et al., 1987; Baumann and Kuhl, 2002; Bolte et al., 2003; Fredrickson, 2004).

### Positive mood facilitates cognitive control processes involved in task switching and response selection

In addition to broadening attention and promoting access to distant semantic associations, positive mood also facilitates creative problem solving by facilitating task switching and response selection. The remote association task (RAT) was created by Mednick (1962) to investigate specific aspects of cognitive control and creative problem solving. The RAT consists of presenting three seemingly unrelated words that are associated with a third solution word in different ways. For instance, the words: TENNIS, STICK and SAME are associated with the solution word MATCH by formation of a compound word (matchstick), by semantic association (tennis match), and by synonymy (same = match). The RAT task requires both broad divergent thinking and more focused convergent thinking. During broad divergent thinking, solvers need to access a wide set of associations (i.e., association words for TENNIS include: GAME, RACKET, BALL, COURT, SET, PLAYER, MATCH). During focused convergent thinking, solvers need to integrate distant semantic associations in different ways to find the correct meaningful solution (i.e., of all the associations, only MATCH fits with the words: TENNIS, STICK and SAME). Several groups have shown that on Mednick's creative RAT, participants in a positive mood state produced more solutions compared to controls in a neutral or negative mood, both in a sample of college students as well as in practicing physicians (Isen et al., 1987; Estrada et al., 1994; Rowe et al., 2007).

We modified Mednick's (1962) RAT task into a simpler version called the Compound Remote Association (CRA) task in which the solution word has to form a compound word or phrase with each of the three target words (Subramaniam et al., 2009). In the CRA task, the solver is given 3 words such as “tooth,” “potato,” and “heart” and is asked to think of a fourth word that can be combined with all three to form a compound word or phrase. The solution word can be placed either before or after any of the three words in the triad to form the compound word. In this case, “sweet” is the solution word, which forms the compounds “sweet-tooth” “sweet-potato,” and “sweetheart.” In the CRA task, when participants see the word “tooth” they need to inhibit the dominant association “ache” or “pain” when they realize that pain does not fit with potato (i.e., “potato-pain?”), and must switch to select an alternative non-dominant solution (i.e., “sweet” forming sweet-tooth, sweet-potato, and sweetheart). These problems are typically associated with insightful solutions, in which participants have an “Aha!” or “Eureka” experience during solution success.

Insightful problem solving requires engagement of cognitive control mechanisms involved in task switching and alternate response selection because there is frequently an impasse where solvers feel stuck, and believe they are making no progress toward the solution. To overcome this impasse, solvers need to inhibit prepotent albeit irrelevant associations in order to allow access and integration of remote yet solution-relevant concepts. Solvers are then able to suddenly recognize connections that had previously eluded them, and instantly see the solution in a new light in the “Eureka!” experience. Therefore, insightful problem-solving requires inhibition of task-irrelevant but prepotent responses, as well as task-switching to broader but non-dominant solution-relevant associations (Subramaniam et al., 2009).

We found that healthy college students higher in assessed positive mood (assessed with the Positive Affect Negative Affect Scale immediately prior to the experiment) solved more CRA problems overall, and specifically solved more CRA problems with insight, compared to college students lower in assessed positive mood. We also found that a positive mood was associated with increased brain activity in the medial prefrontal region during a preparatory interval preceding each solved problem. A positive mood was correlated with stronger preparatory signal that led to insight vs. analytical solutions, thus biasing participants toward insightful problem-solving; it appears that increased mPFC activity was associated with greater prefrontal cognitive control, which in turn allowed solvers to switch between broad attentional modes (required for detection of distant remote solution candidates) and focused attention for response selection (required to converge upon the correct solution).

**Figure 2:**
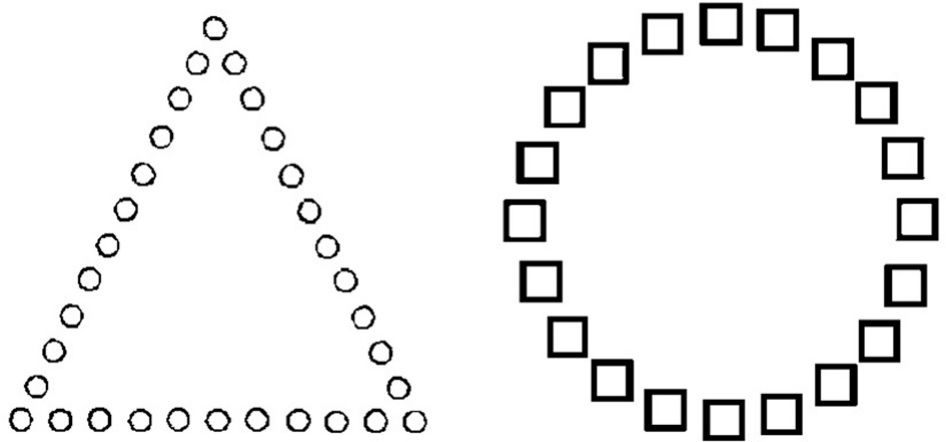
**Global-local task of cognitive control.** For the item on the left, the circle is the target, presented on a local dimension. For the item on the right, the target circle is presented on a global dimension [reproduced with permission from Baumann and Kuhl (2005)]. Participants in a positive mood demonstrated increased cognitive control by responding to non-dominant local features (i.e., detecting the target circles that made up the triangle).

Consistent with this interpretation, Baumann and Kuhl (2005) demonstrated that participants in a positive mood showed greater cognitive control in a global-local shape detection task (see Figure 2). In Figure 2, for the item on the left, the target “circle” is presented on a local dimension, but is presented on a global dimension for the item on the right. In order to induce the target mood state, participants were asked to recall personal experiences that felt positive or negative, and were then asked to note down positive, neutral, and negative words which reminded them of their past experiences. Participants were then given a self-generated prime-word (i.e., either positive, neutral or negative), which was then followed by the global-local shape task. When participants were in a positive vs. a neutral or negative mood, they responded faster to targets presented on a local dimension when the task necessitated local-feature detection. As such, they showed increased cognitive control by overcoming a global precedence in order to respond to non-dominant local features (i.e., detecting the circles that make up the triangle). In contrast, participants in a negative mood had slower response times to local targets when compared to a neutral mood. These results suggest that participants in a negative mood state had less cognitive flexibility, and thus were slower in switching to a non-prepotent local mode of processing from the default global mode of processing.

Switching can occur between global and local modes of attention, and also between strategies (Dreisbach and Goschke, 2004; Baumann and Kuhl, 2005). Prior research has shown that a positive mood enhances switching between different perspectives (Isen and Daubman, 1984; Isen, 2005). A shift from a focused local to a global attentional state increases the scope of semantic and visual access, which can promote enhanced selection of solution-relevant responses on classic insight problems such as Duncker's candle task (Duncker and Lees, 1945). Participants watched either a comedy film clip or a neutral film clip to induce the target mood state, followed by a word-rating scale to verify the efficacy of the mood manipulation. Following the manipulation check, participants were asked to solve Duncker's candle task. In this task, participants were presented with a box of tacks, a candle, and a book of matches and were asked to attach the candle to the wall in a way that it will burn without dripping wax on the table or floor. Participants in a positive vs. neutral mood were better able to shift their perspective from viewing the box as a container to viewing the box as a platform for the candle. Switching away from the prepotent view of perceiving the box as a container to viewing it as a platform facilitated solving when participants realized that the upright candle could be tacked from the matchbox to the wall (Isen et al., 1987). More recently, a positive mood has been shown to reduce perseveration and facilitate switching/shielding from distracters (Dreisbach and Goschke, 2004). It is important to note, however, that an optimal balance between task switching and task-shielding operations must be maintained in order to maximize creative problem-solving. A field of attention that is too broad, with far-ranging associations and a high degree of switching between solving strategies, can lead to increased distractibility, inhibiting solution selection. Similarly, a solver who maintains a very focused and narrow spotlight of attention, with little ability to switch away from unsuccessful strategies, will become “stuck” in a fruitless search for the solution.

## Medial prefrontal neural activation is a key component of the influence of positive mood on cognitive processing

While a significant amount of research has been conducted on the neurobiology of reward (e.g., Beninger, 1991; Phillips et al., 1992; Knutson and Cooper, 2005; Cooper et al., 2009) and on the neural correlates of positive mood (e.g., Lane et al., 1998; Damasio et al., 2000; Habel et al., 2005; Burgdorf and Panksepp, 2006), only few studies have examined the neural system activation patterns mediating positive mood and cognition (Federmeier et al., 2001; Subramaniam et al., 2009, 2013).

Federmeier et al. (2001) used ERP to investigate the influence of positive mood on semantic relatedness. Mood was manipulated in each participant using positive and neutral photos from the International Affective Picture System. The effectiveness of the mood state induction was assessed with a written questionnaire asking participants to report on how much they liked the photos from each condition. Federmeier et al. (2001) found that a positive mood facilitated meaningful processing of distantly-related information, thus broadening semantic access and integration. Specifically, these investigators examined the N400 response, as an index of semantic relatedness on a sentence completion task. Participants read sentence pairs ending with either: (1) the most expected word, (2) an unexpected word from an expected semantic category, or (3) an unexpected word from a different (related) category. An example of a sentence was: “They wanted to make the hotel look more like a tropical resort. So, they planted rows of … ” For the final word, (1) the most expected item, would be “palms”; (2) an unexpected item from the same semantic category would be “pines”; (3) and an unexpected item from a different semantic category would be “tulips.” Following a positive mood induction vs. a neutral mood induction, participants showed a reduced N400 amplitude (indexing meaningful processing) for unexpected different category exemplars. Thus, a positive mood compared to a neutral mood specifically facilitated meaningful processing of distantly-related between-category exemplars. Subsequent studies have replicated the impact of positive mood on broadening the scope of semantic access (Rowe et al., 2007; Subramaniam et al., 2009). This suggests that a positive mood may facilitate integration processes by relaxing constraints to enhance both semantic distance as well as breadth of attention.

**Figure 3:**
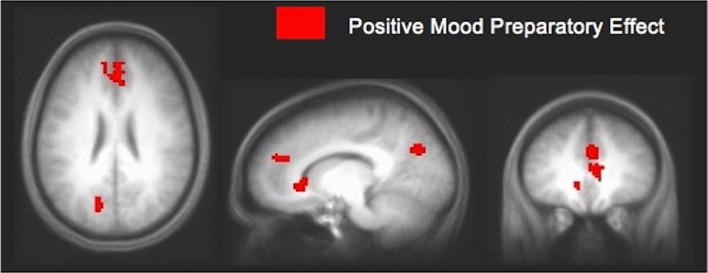
**Positive mood preparatory effect.** Signal change in mPFC/ACC and posterior cingulate cortex during the preparation period for participants high in positive mood vs. participants low in positive mood, at a threshold of *p* < 0.005 (uncorrected). Increased mPFC signal enhanced cognitive preparation, predisposing positive mood participants to solve more problems overall, and to specifically solve more creatively with insight [adapted from Subramaniam et al. (2009)].

In an fMRI study using Compound Remote Associate (CRA) problems (Subramaniam et al., 2009), we found that participants who were higher in a positive mood state (assessed with the PANAS, immediately prior to the fMRI study) revealed greater ACC/mPFC activity during a preparation period prior to problem onset when compared to participants lower in a positive mood (see Figure 3). Increased mPFC activation prior to problem onset appears to represent an enhanced state of creative cognitive preparation, as it correlated with participants' overall solving ability, with insight solving and with positive mood. These data suggest that mPFC preparatory signal predisposed positive mood participants to solve more problems overall, and to specifically solve more of the problems using insight (Subramaniam et al., 2009). We also demonstrated, using EEG topography and frequency recordings that insight preparation (i.e., preparatory brain states that bias and facilitate subsequent solving with insight), compared to step-by-step analytical preparation, involved increased activity over medial prefrontal regions (Kounios et al., 2006). Increased activation over medial prefrontal regions appeared to reflect increased preparation to exert top-down cognitive control mechanisms to switch cognitive strategies and select the correct solution. Additionally, insight compared to step-by-step analytical preparation also involved occipital alpha activation (which inversely relates to neural activity) thought to shield top-down processes from interference by bottom up visual stimulation (Kounios et al., 2006). Thus, a positive mood may predispose and facilitate creative cognition by enhancing activation within the medial prefrontal cortex, thus supporting the regulation of attention/cognitive control processes as the demands of the task necessitate. Creative insightful solutions may be facilitated by modulating the shift between task shielding effects (i.e., shown by visual occipital alpha activation during insight preparation) and task switching/selection effects (i.e., shown by increased activity over medial frontal regions). Enhanced cognitive control induced by a positive mood enables solvers to switch away from task-irrelevant distracters in order to select from a broader array of solution-relevant information.

In a subsequent fMRI study, we demonstrated that not only a positive mood, but also positive stimuli could enhance neural patterns associated with creative cognition (Subramaniam et al., 2013). We specifically examined how positive stimuli (i.e., positive words) could modulate creative metaphoric processing. A metaphor is a figure of speech in which a subject is compared to an unrelated object (i.e., *He is the apple of my eye*). Metaphoric comprehension is considered a type of creative cognition, and involves linking previously unassociated meanings in order to create a new understanding of one item in terms of another (in the example above, the “apple” refers to someone beloved). According to the Gradient Salience Hypothesis (Giora, 1997, 2003), metaphoric processing is modulated by the conventionality, frequency, and familiarity of the words. We investigated neural processes associated with conventional (familiar) metaphors (e.g., *brain freeze*) as well as novel (unfamiliar) metaphors (e.g., *unfenced idea*). Conventional metaphoric processing involves recalling a familiar closely-connected meaning (Amanzio et al., 2008). By contrast, novel metaphoric processing is a type of creative cognition that involves formulating new meanings from unfamiliar expressions that have not been previously encountered (Subramaniam et al., 2012a).

In our metaphor study (Subramaniam et al., 2013), prior to fMRI scanning, participants received a list of word pairs (novel unfamiliar metaphors, conventional familiar metaphors, and unrelated meaningless words) and were asked to denote the valence (positive, negative, or neutral) of each word pair. For example, conventional familiar metaphors such as “beautiful mind,” “visual field,” and “sour grapes” were typically classified as having positive, neutral and negative connotations, respectively. Similarly, novel unfamiliar metaphors such as “joy bits,” “memory phantoms” and “caged cry” were also typically classified as having positive, neutral, and negative connotations. During fMRI scanning, participants had to decide whether the word pairs formed meaningful or meaningless expressions.

**Figure 4:**
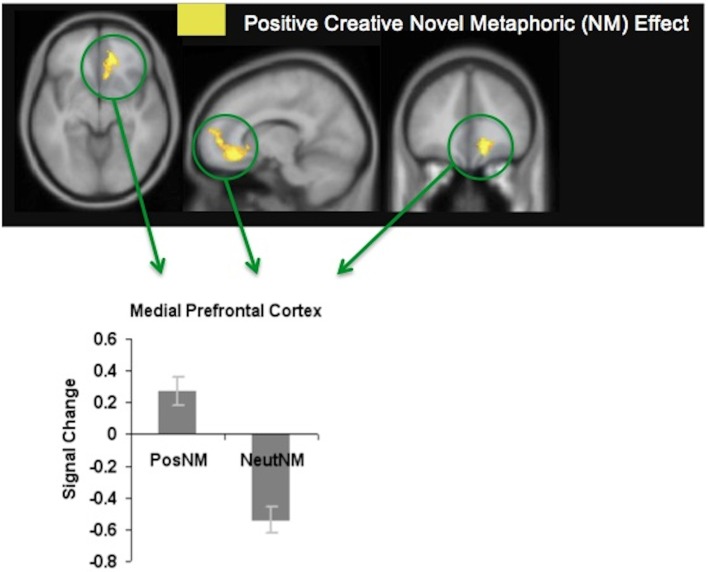
**Positive creative novel metaphoric effect.** Signal change for positively-valenced creative novel metaphors (PosNM) vs. neutral novel metaphors (NeutNM) at a threshold of *p* < 0.001 (uncorrected). Mean mPFC signal (beta weights) for each condition is revealed in the bar chart [adapted from Subramaniam et al. (2013)].

We found that participants made more accurate meaningful judgments when viewing metaphors that were positively-valenced, and that this was associated with enhanced modulation of the mPFC. The mPFC was activated during positively-valenced metaphors in general, but revealed most activation specifically when participants viewed positively-valenced novel metaphors (see Figure 4). We hypothesize that increased mPFC signal for positively-valenced novel metaphors (which may also have induced a positive mood reaction) enabled participants to have a broader scope of attention that facilitated access to a broad array of different possible semantic interpretations for the unfamiliar word pairs. Greater mPFC modulation may also have enabled participants to exert greater cognitive control to eliminate, or switch away, from meaningless interpretations. In this way, participants were better able to select the correct interpretation that linked the novel words together in a meaningful way.

We do not mean to imply that the observed activations in the medial prefrontal cortex (mPFC) represents a neural correlate of positive mood, nor that positive mood states will always benefit cognition. We also do not mean to suggest that the mPFC is the only brain region which mediates positive mood-cognition interactions. Indeed, other regions such as the basal ganglia—that are central to both cognition (McNab and Klingberg, 2008) and reward processes (Knutson and Cooper, 2005)—are also important in mood-cognition interactions. However, our data do indicate that positive mood enhances activity in the rostral dACC/mPFC, and that increased activation in this region (even prior to the onset of a problem) is significantly correlated with improved creative cognitive solving abilities.

## Dopaminergic neuromodulation plays an important role in the relationship of positive mood to cognition

Some of the earliest investigators in this field proposed that the cognitive effects of positive mood were likely to be mediated by increased dopaminergic release in the brain (Ashby et al., 1999, 2002). Ashby et al.'s (1999, 2002) predictions were informed by research on the neurobiology of reward processing, which is associated with increased positive mood and phasic release of dopamine (Beninger, 1991; Bozarth, 1991; Phillips et al., 1992; Schultz, 1992). They predicted that increased dopamine tone in prefrontal cortex induced by a positive mood would enhance access to a broad range of associations and facilitate switching between attentional modes/strategies. Indeed, prior research suggests that such increases of dopamine in the prefrontal cortex up-regulate cognitive control mechanisms (Braver et al., 1999). However, we must emphasize that the relationship between positive mood, reward processing, dopamine neuromodulation, and cognitive control is likely to be highly non-linear and complex. Studies from animals and humans reveal that the prediction and receipt of rewards is associated with phasic activation of midbrain dopaminergic firing, while a positive mood is associated with overall increased prefrontal dopamine release each with distinct implications for cognition (Ashby et al., 2002; Schultz, 2002; Montgomery et al., 2007). As this field is still in its early stages, we present here only a brief overview and a proposed model for the role of dopaminergic modulation in the cognitive effects of positive mood, in order to stimulate further research.

First, while a positive mood facilitates creativity on tasks that require divergent (broad) thinking, it is important to note that Rowe et al. (2007) demonstrated that a positive mood may not be beneficial for tasks requiring focused attention. This finding raises the question of whether there is an optimal mood specific to the demands of the task at hand. Specifically, Rowe et al. (2007) used positive and sad music to induce positive and negative mood states, relative to the neutral condition (which was induced by participants reading facts about Canada); a mood manipulation check demonstrated that the mood induction was successful. Participants were then tested on the Eriksen flanker task in which they had to selectively attend to the central letter while ignoring distracting flanker stimuli. Rowe et al. (2007) found that positive mood states increased participants' visual scope of attention, making them more susceptible to the flanker distracters. Participants who had greatest visuospatial breadth (increased susceptibility to the flanker distracters) also showed greatest breadth in semantic scope of attention (indexed by the number of remote associates accessed on the Remote Associates Task). Thus, while a positive mood does broaden attention both in terms of internal conceptual semantic associations and external visual space, it does not necessarily improve all aspects of cognitive performance.

Chermahini and Hommel (2012) investigated this question more closely by examining the relationship between positive mood, creativity, and dopamine on a creative divergent thinking task. They found that performance on a divergent thinking task was associated with spontaneous eye-blink rates (an indirect measure of an individual's central dopamine tone), following an inverted U-shape. The results suggest that greatest cognitive flexibility is reached at an optimal level (“sweet spot”) of dopamine release. They next used mood manipulation techniques in conjunction with eye-blink rates (EBR) on this task to study the influence of a positive mood on EBR and creative divergent thinking. Mood was manipulated through mental recall in which participants were asked to write down and recall positive and negative personal events in their life that made them happy or sad. The mood manipulation check confirmed that participants were more happy after the positive mood induction than before, and were less happy after the negative mood induction than before. EBRs were recorded right after the mood induction. Participants then performed an Alternate Uses Task used to assess divergent thinking. In this task, they were given a household item such as a cup, and were asked to write down as many possible uses for it in 5 min.

The investigators found that the positive mood induction increased EBR and increased flexibility on the divergent thinking task compared to baseline. The more positive participants became, the more flexible they were on the divergent thinking task and the more their EBRs increased. These results indicate that the changes in EBR (indexing central dopamine levels) induced by a positive mood were related to the increases in flexibility. This relationship was not found for the negative mood condition. Interestingly, upon closer inspection, when the investigators conducted a median split of EBR at baseline, they found that the induction of a positive mood improved flexibility in the low-EBR group only. These data indicate that the relationship between positive mood, dopamine tone, and creative problem-solving is non-linear, and suggest that positive mood induction methods might have the most cognitive impact on individuals who have low basal dopamine tone.

**Figure 5:**
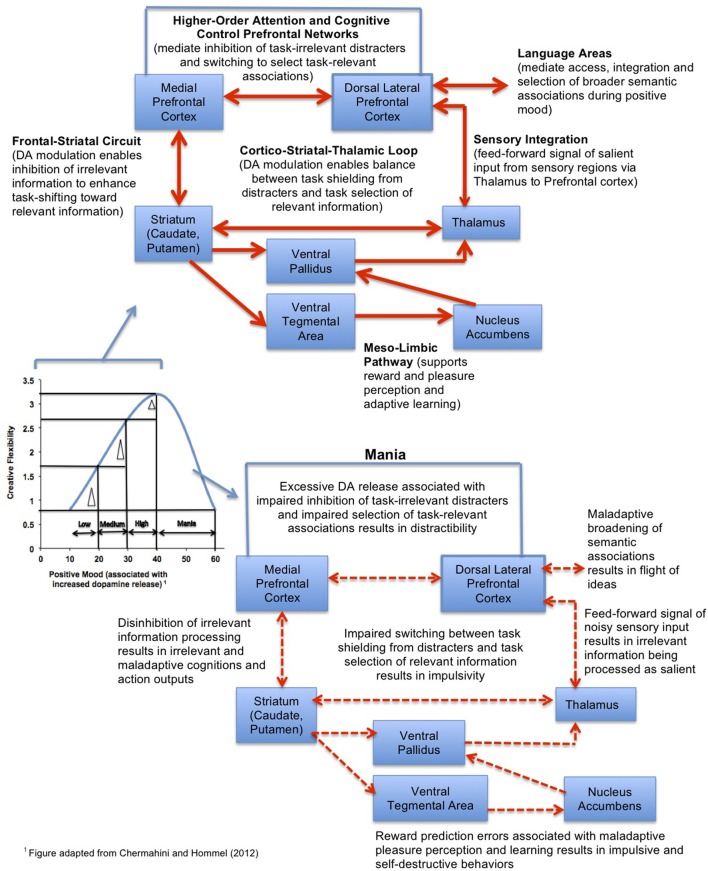
**Model of the neural system interactions mediating the effect of positive mood on creative cognition.** The model predicts that the largest gains in cognitive flexibility (indicated by the delta triangle) induced by positive mood inductions will be observed for subjects with low-medium basal positive mood levels, while individuals with mania will have impaired creative flexibility.

To extend upon Chermahini and Hommel's (2012) study, we propose a working model (see Figure 5), in which positive mood induction methods would have the greatest cognitive benefit on tasks which require a broad scope of attention and particularly for individuals with low-medium basal positive mood levels (and also, theoretically, lower prefrontal dopamine tone), moving these individuals toward an optimal level of cognitive flexibility. In individuals with high basal positive mood levels, the cognitive benefit will be smaller. In light of the evidence so far, we tentatively propose that positive mood inductions associated with increased dopamine release in the prefrontal cortex will help the lower-mood individuals achieve improved cognitive control operations related to task-switching and response selection (Cohen and Servan-Schreiber, 1992; Cohen et al., 1996), which are essential components of creativity. These beneficial cognitive effects will be maximized on tasks that require a broader scope of attention (Dreisbach and Goschke, 2004; Rowe et al., 2007).

By contrast, positive mood inductions in individuals with abnormally high states of elevated mood, such as occurs during a manic episode, would be contraindicated, as this would contribute to further impairment in already impaired cognitive control processes (Figure 5). Individuals suffering from mania are known to show weakened prefrontal control (Morice, 1990; Johnson, 2005), and it is theorized that hyperdopaminergic tone in prefrontal cortex during mania is associated with disinhibition in fronto-striatal efferents, contributing to a “noisy signal” that impairs the selection of task-relevant information (Swerdlow and Koob, 1987; Diehl and Gershon, 1992). Noisy signal from fronto-striatal efferents into the mesolimbic pathway contributes to reward-prediction errors, such that manic individuals have difficulty distinguishing behaviorally salient and adaptive rewarding stimuli from less salient stimuli (Abler et al., 2008). Consequently, mania is associated with an overly broad scope of attention, “hyper-salience,” distractibility, flight of ideas, and impaired decision-making (Clark et al., 2002)—suggesting again that there is a U-shaped curve—“too much” dopamine tone in prefrontal regions can be as deleterious to adaptive creative problem-solving as too little (Murphy et al., 1999, 2001). In this light, it is interesting that mania—at least in its early phases of hypomania—is characterized by broad and creative semantic associations and thought processes, as well as high creative output, suggesting that there may be an optimal level of prefrontal dopamine release required for maximal creative performance (Schuldberg, 1990; Jamison, 1993; Kaufmann, 2003) (Figure 5).

## Behavioral interventions can be used to improve positive mood and enhance cognition

A number of behavioral interventions have been developed from the “positive psychology” model, and include: practicing optimistic thinking, increasing attention and memory of positive stimuli and experiences, practicing mindfulness and acceptance, and increasing socializing behaviors. Sin and Lyubomirsky (2009) conducted a meta-analysis of 49 positive psychology interventions, and found that they were highly effective in enhancing positive mood and overall well-being, with a medium effect size of.29. Additionally, such positive psychology interventions enhance mood and well-being not only in healthy participants, but also in a range of patient populations, including those suffering from depression, anxiety, schizophrenia and HIV (Fava et al., 2005; Seligman et al., 2006; Moskowitz et al., 2012; Meyer et al., 2012; Caponigro et al., 2013). While the cognitive and underlying neural effects of these interventions are not yet known, this represents a fruitful area of future investigation.

In the past few years, a novel approach to positive mood interventions has emerged which allows for participants to volitionally up-regulate positive mood states via neurofeedback techniques such as real-time fMRI. Participants can be trained to voluntarily control neural fMRI signal from target regions implicated in positive emotional processes (Johnston et al., 2010, 2011). Though it has not yet been studied, positive mood up-regulation using neurofeedback is likely to modulate cognition, particularly creative cognition. Based on our prior findings (Subramaniam et al., 2009), we would predict that participants who are trained to up-regulate signal within mPFC and bilateral lateral prefrontal cortices will show enhanced positive mood preparatory brain states. These enhanced positive mood preparatory brain states are likely to facilitate attention/cognitive control processes on creative tasks.

It is possible that real-time fMRI neurofeedback techniques could be used to enhance positive mood and cognition not only in healthy individuals, but also in individuals with depression and schizophrenia who are characterized as having both reduced sensitivity for maintaining positive affect and experiences in memory as well as heightened sensitivity to negative stimuli (Sloan et al., 1997; Kan et al., 2004; Gard et al., 2011; Holt et al., 2011; Ursu et al., 2011). Interestingly, in patients with depression and schizophrenia, impaired attention, memory, self-regulation and cognitive control associated with reduced cingulate/prefrontal activation, has shown to be reversed with stimulation or enhanced activation in these regions (Pascual-Leone et al., 1996; Mayberg et al., 2005; Haut et al., 2010; Subramaniam et al., 2012b). Thus, it is possible that real-time fMRI neurofeedback targeting the anterior cingulate and medial prefrontal cortex may enhance positive mood-cognition interactions in such patient groups.

## Several caveats

While we have argued in this review that positive mood exerts some specific effects on neural activation states, in turn influencing cognitive processes, it is important to stop and consider whether other possible mechanisms might explain the effects of positive mood on cognition. For example, could the cognitive-enhancing influence of a positive mood be simply due to the non-specific effects of physiologic arousal? To test this, Isen et al. (1987) performed a study that included participants engaging in exercise (to generate arousal with no affective tone), along with a positive mood group, a neutral mood group, and a negative mood group. This study found that participants in the exercise condition did not demonstrate more solutions on classical insight problems, such as Duncker's candle task, compared to the neutral or negative mood condition; in contrast, the positive mood group produced more solutions than any of the other groups. These findings suggest that the influence of positive mood on cognitive control/flexibility is not simply due to physiologic arousal.

Other investigators have proposed that a positive mood facilitates a more heuristic, superficial mode of processing. For instance, positive mood participants have been suggested to use “satisfising” and superficial, rather than optimizing solving strategies (Kaufmann and Vosburg, 1997). Estrada et al. (1997) found evidence to the contrary while observing physicians in a positive mood state decide on a diagnosis for a rare case of liver disease. Physicians in this study showed no evidence of superficial processing, defined as deciding on a diagnosis without having enough evidence. Furthermore, we have argued previously that superficial processing induced by a positive mood would lead to more premature and incorrect responses; yet, high and low positive-mood participants made equally few errors on our creative problem-solving task (Subramaniam et al., 2009). Therefore, it appears that the nature of the task is important, and that one cannot make generalized conclusions about mood-cognition interactions without taking the specific task and event/contrast of interest into account. It is possible, for example, that positive mood participants may engage in more superficial processing on certain tasks compared to neutral mood participants simply because they find the task boring or incompatible with their mood state. Finally, some researchers have proposed that a positive mood reduces overall cognitive capacity (Mackie and Worth, 1989). For instance, a positive mood may increase the load on working memory because it increases the occurrence of positive mood-related thoughts that are more likely to interrupt cognitive processing (Seibert and Ellis, 1991). The evidence for this assertion does not appear to be strong, however; and again, it is likely that the demands of the task (selective focused attention vs. broad task-switching or interference resolution) play an important role in determining the ultimate cognitive outcomes of a positive mood state.

Another caveat in terms of the existing literature is the chicken-and-egg question of which mechanisms are primary and which are secondary. Does adopting a positive mood state induce increased dopaminergic tone in prefrontal regions and enhance access to distant semantic associations? Or do individuals with broader and more rapidly accessible semantic association networks tend to exhibit positive mood states? Or are both of these phenomena— a certain *joie de vivre* plus rapidly spreading access in semantic networks—the manifestation of some other primary underlying neural signature (e.g., dopaminergic tone in striatal and prefrontal neural systems)? Only carefully designed mood-induction studies, as well as probes into mood, reward responsivity, and creative problem-solving across different behavioral phenotypes, can answer these questions.

We also need to add a word of caution about potential clinical applications. It may very well be the case that inducing a positive mood with the goal of enhancing more far-ranging semantic associations could be deleterious in the case of people predisposed to mania or to schizophrenia. In the former, one might precipitate an actual manic episode (as can happen when such people are treated with antidepressants). In the latter, one might worsen symptoms of thought disorder, since some forms of schizophrenia are in fact characterized by the presence of too many broad and maladaptive associations. Sometimes, too much “creativity” can be deleterious.

It is highly likely that people can learn—through a variety of methods—to increase their positive mood volitionally, using a range of straightforward behavioral techniques, and also through real-time fMRI neurofeedback. The question will be: For whom are these approaches most beneficial? And how should they be combined with other approaches in order to maximize healthy functioning and well-being? Enhancing broader semantic associations and greater cognitive flexibility may be very useful for a person with cocaine addiction who is in an active treatment program learning new critical psychosocial skills, but may be very maladaptive for the same individual when they are in their “using” environment. A depressed individual with low motivation and many perseverative ruminations about past failures may benefit greatly from mood enhancement that broadens their scope of attention and facilitates examining alternative interpretations through cognitive behavioral therapy. But absent such psychological treatment, the same broadening of attention may lead the depressed individual to contemplate various methods of self-harm/suicide as a viable alternative.

## Some happy thoughts for the future

We have entered a new era in our understanding of how to optimize learning in educational environments, and interestingly, it is an era that recognizes the importance of developing creative problem-solving capacities, rather than simply drilling the rote memorization of facts and figures. We now understand that critical learning occurs best in social contexts, under conditions of high intrinsic motivation, and is facilitated by appropriate rewards—all conditions that generate and sustain positive mood states. In the coming years, we can look forward to an even more knowledgeable and nuanced grasp of how to use these positive moods to help learners develop their skills as innovative and creative problem-solvers.

Similarly, there is a sea change in our approach to facilitating health and well-being in individuals with psychiatric illness. In the past decade, the field of mental health treatment has embraced a model of autonomy, dignity, and recovery for people with psychiatric illness; this has generated a growing emphasis on preventive interventions, on the techniques of positive psychology, and on developing therapies that go beyond the palliation of symptoms and that instead generate optimal well-being and community functioning. With a new focus on the important role that expectation and motivation play in the recovery process, and with a greater understanding of the many behavioral elements that can induce positive changes in mood state, we can expect to become even more refined in our ability to create mood-enhancing interventions to help individuals engage in adaptive problem-solving and creative skill-learning and coping.

Finally, the nascent but rapidly growing area of computerized cognitive training for psychiatric illnesses is moving in the direction of “gamification”—adapting the techniques of game developers and interactive software experts to make the cognitive training as engaging, fun, and rewarding as possible (Vinogradov et al., 2012). Such a treatment approach, if it reaches its full potential, will be able to harness the mood-elevating aspects of well-designed games and of social networking to promote the highest possible degree of cognitive enhancement and learning. The ultimate aim will be to help all individuals maximize their “happiness” and “creative problem-solving” potential, so they can—to paraphrase Lincoln—make up their minds to be as happy and successful as possible.

### Conflict of interest statement

The authors declare that the research was conducted in the absence of any commercial or financial relationships that could be construed as a potential conflict of interest.
